# Investigating Strategies for Sustaining Modern Health Information Systems for Enhanced Care Delivery in Fragile Resource-Constrained Settings Through Electronic Medical Record Implementation in Haiti: Qualitative Study

**DOI:** 10.2196/82396

**Published:** 2026-07-27

**Authors:** Benito D Isaac, Annie Michaelis, Meredith Casella Jean-Baptiste, Loune G Viaud, Wesler Lambert

**Affiliations:** 1 Department of Global Health and Social Medicine, Harvard Medical School, Harvard University, 641 Huntington Avenue, Boston, MA, 02115, United States, 1 (617) 432-1707; 2 Partners In Health (PIH), Boston, MA, United States; 3 Zanmi Lasante (ZL), Port-au-Prince, Haiti

**Keywords:** digital health, electronic medical records, Haiti, low- and middle-income country, LMIC, health information systems, OpenMRS, qualitative methods

## Abstract

**Background:**

Robust and reliable health information systems (HISs) are foundational to equitable health care delivery in resource-constrained settings. Yet, HISs often exhibit significant fragmentation and complexity, which stem from many factors, including inadequate infrastructure, limited and unevenly allocated financial resources, expertise gaps, and a lack of integrated systems. At the same time, advances in modern HISs and digital technologies, such as electronic medical records (EMRs), present opportunities for addressing these limitations and supporting evidence-based health systems if well implemented and sustained. However, limited attention has been paid to how modern and resilient HISs can be effectively sustained in fragile, resource-constrained settings.

**Objective:**

This empirical study seeks to identify pathways for strengthening the resilience, adaptability, and contextual-fit of EMRs in fragile, resource-constrained settings such as Haiti.

**Methods:**

Using a qualitative research methodology, the study used semistructured interviews with purposive sampling of key informants, including frontline doctors, nurses, and IT or data specialists. Interview participants were selected for their expertise and capacity to offer insightful and varied viewpoints on the topic. Transcripts were analyzed using an inductive approach to identify key emerging themes.

**Results:**

The findings of this investigation reveal that implementing resilient EMR in limited-resource settings, such as Haiti, requires a comprehensive strategy that accounts for ecosystemic challenges from interdependent systems (electricity, sociopolitical instability, internet connectivity), as well as the intrinsic complexities of legacy systems.

**Conclusions:**

Drawing on empirical evidence, the study identifies a set of protective strategies that, if effectively implemented, may enhance the resilience and adaptability of EMRs. These strategies include prioritizing integration and interoperability between systems, building redundancy to mitigate cascading failures derived from other interdependent systems, strengthening staffing to support system use, right-sizing the paper footprint (eg, improving handwriting-to-text scanning and digitization solutions), and embracing technological innovations.

## Introduction

Robust and reliable health information systems (HISs) are foundational to equitable health care delivery in resource-constrained settings [1]. High-functioning HISs enable timely, evidence-based decision-making across all levels of the health system—from clinical decision support to broader program and policy management—while improving workflows and resource allocations [2]. Yet, HISs in such settings often exhibit considerable fragmentation, complexity, and lack of interoperability, stemming from factors that include, but are not limited to, inadequate infrastructure [3], limited financing [4-7], expertise gaps, lack of integrated enterprise systems, and uneven endowment of resources across geographical areas [8,9]. These constraints hinder the ability of HISs to effectively support health care delivery by undermining data quality, disrupting workflows, and weakening continuity of care with consequences for patient outcomes, service efficiency, and decision-making [9,10].

Concurrently, advances in modern HISs and digital technologies, such as electronic medical records (EMRs), present opportunities for addressing these limitations and supporting evidence-based health systems if well implemented and sustained. Existing literature on the topic has primarily focused on describing the main characteristics of HISs in low- and middle-income countries (LMICs) and identifying common barriers and enablers of EMR implementation in such contexts. Yet, less attention has been paid to how EMR systems evolve and are sustained over time in fragile health systems where successful implementation is shaped not only by technical design but also by political and social instability, competing stakeholders’ priorities, chronic resource constraints, and the persistent gap between the promise of modern HISs and their practical realization.

Haiti provides a particularly important case through which to understand these dynamics. In the early 2000s, the introduction of an EMR platform for health care data management in a Haitian public hospital, though limited in scope, marked a significant milestone in the country’s digital health development. Initially launched as part of the multidrug-resistant tuberculosis and HIV program, this pioneering initiative improved care for patients with HIV in the country [11] and contributed to the later development of OpenMRS [12], an open-source EMR platform widely adopted by governments, nonprofit organizations, and other health care institutions globally. Therefore, this experience offers a concrete and valuable opportunity to understand how modern platforms are introduced, adapted, and maintained in fragile, resource-constrained settings.

Drawing upon sociotechnical theoretical frameworks and empirical evidence, this study seeks to address this critical gap by integrating the perspectives of data or IT experts and frontline health care providers to identify pathways for strengthening the resilience, adaptability, and contextual-fit of EMRs in complex, resource-constrained settings such as Haiti.

## Methods

### Study Design

This study used a qualitative research design centered on in-depth, semistructured interviews with key informants, selected through purposive sampling based on their relevant expertise, professional proximity to EMR implementation, and presumed ability to provide well-informed and diverse perspectives on the subject matter.

### Study Setting

The study was conducted within 2 public health care facilities operated by the Haitian Ministry of Health (MSPP) and supported by the Haitian health care nonprofit organization, Zanmi Lasante (ZL; the sister organization of the global health nonprofit, Partners In Health). The 2 facilities were selected as information-rich cases that capture meaningful variation in resource endowment, thereby improving the transferability of the findings to comparable LMIC settings. One of these facilities is a tertiary referral teaching hospital, Hôpital Universitaire de Mirebalais (HUM), and the other is a primary health care center, Saint-Michel Health Center of Boucan-Carré. These health care facilities are located in the communes of Mirebalais and Boucan-Carré, respectively, both within the rural Central Plateau department of Haiti.

### Sampling and Recruitment

The researchers used a purposive sampling method to select study participants based on job function, tenure at ZL or Partners In Health, and location of work to ensure diversity across geographic locations and relevant professional backgrounds. An equal number of health care providers were chosen from both HUM and Boucan Carré study sites. Information systems specialists with extensive experience working for the institution both in the Partners In Health Boston office and at HUM, Boucan Carré, and the ZL central office in Haiti were also included, thereby providing a varied range of perspectives and expertise.

### Data Collection

An in-depth interview guide was developed based on study objectives and relevant literature. It included open-ended questions with the flexibility to elicit participants’ experiences and reflections about the topic. The guide was tested and adapted during the design process. The participants in the test phase were excluded from the final data collection. Key informant interviews were completed in-person or through virtual video conference meetings. To ensure confidentiality, both in-person and virtual interviews were conducted in private rooms. The duration of interviews varied, ranging from approximately 45 minutes to 1 hour and 30 minutes. Interviews were conducted in English or Haitian Creole, contingent upon the native language and preference of each participant. Interviews were conducted until thematic saturation was achieved, defined as the point at which additional interviews no longer generated new themes or substantially enriched analytical perspectives.

### Analysis

A research assistant, trained by the principal investigator (PI) in ethical standards and research confidentiality, transcribed the Haitian Creole interviews. The English interviews were conducted remotely via the Zoom app with the transcription feature activated; the transcripts were later reviewed and edited by the PI for accuracy and spelling errors. The PI, who is fluent in English and a native speaker of Haitian Creole, completed all translation tasks from Haitian Creole into English for this research. Prior to analysis, all transcripts were carefully reviewed to ensure accurate translation and fidelity to the original interview audio recordings. The PI also reviewed all automatically generated Zoom transcripts and corrected any inaccuracies.

Using an inductive, thematic approach to content analysis, the interview transcripts were examined to identify emergent themes relevant to the research study. Subsequently, open coding was performed on 4 transcripts to establish preliminary categories for analysis, providing a foundation for the extraction of key themes. The remaining transcripts were then coded through an iterative process that allowed for continuous reexamination and deepening of codes and emergent themes. We used Dedoose (version 9.05; cloud app for managing, analyzing, and presenting qualitative and mixed method research data) for the data analysis.

Given the PI’s insider familiarity with the context, reflexive memo-writing and attention to discrepant cases were used to limit the influence of prior assumptions.

### Ethical Considerations

This study obtained institutional review board (IRB) approval from both Harvard University IRB (CR22-0157-01) in the United States and ZL IRB (ZLIRB06092022) in Haiti. Verbal consent was obtained from all interview participants. Names and any other identifiable information were redacted from the transcripts to ensure anonymity. All participants in the interviews were adults, and none were classified as members of vulnerable populations.

## Results

### Key Informant Characteristics

In this investigation, a total of 18 key informants agreed to participate in individual interviews. One transcript was removed from the analysis due to issues related to audio and transcription quality. As a result, 17 interviews were included in this analysis (Table 1).

**Table 1.** Number of key informants, by area of expertise and location.

Study site	Health care providers, n (%)	Information system experts, n (%)	Total, n (%)
Hôpital Universitaire Mirebalais	4 (40)	6 (60)	10 (100)
Boucan Carré Health Center	4 (100)	0 (0)	4 (100)
Partners In Health Boston office	0 (0)	3 (100)	3 (100)
Total	8 (47)	9 (53)	17 (100)

### Themes

**Figure 1.**
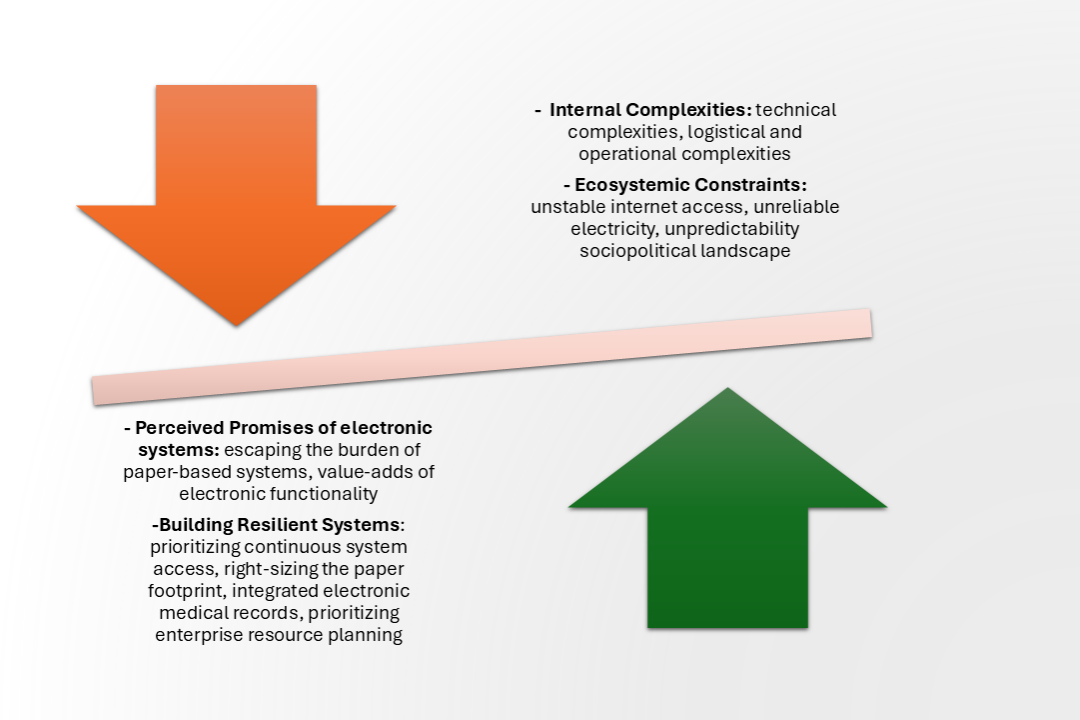
Illustration of the themes derived from the analysis of interview transcripts.

The study findings reveal four primary themes: (1) Perceived promise of electronic systems, (2) Ecosystemic constraints, (3) Internal complexities, and (4) Building resilient information systems (Figure 1).

#### Theme 1: Perceived Promise of Electronic Systems

##### Escaping the Burdens of Paper-Based Systems

Key informants shared a range of perspectives on the current and potential future value of transitioning from paper-based records to EMRs. The most frequently cited reason for investing in the transition to electronic systems was related to the perceived cumbersome and unsustainable nature of paper-based data collection. Respondents noted challenges in finding suitable storage space and maintaining proper management of paper files, resulting in negative impacts on patient care. For example:

I remember once I went to a site and when I got there, I saw a consultation [room] that was supposed to close because the archive was already full and they started putting files in it. And I said to myself, “this is serious, it’s terrible, we have to come up with a plan for paperless.”

[IT or data specialist 5]

In addition to the growing physical burden of storing paper records, key informants often described issues related to patient file loss, lack of privacy in handling patient information, untimely archiving of patient records, and the duplication of patient files. One provider clearly explained the health impacts of such losses:

Okay, if a patient has a lost file, then it has a significant impact because from the first contact you have with the patient, there is a lot of information that you take from when the child is just born to when you see them at the clinic. This information is important because there is information you have since birth. The child may develop a pathology after that you need to link with the birth history*.*

[Provider 1]

##### “Value-Adds” of Electronic Functionality

In addition to describing the ways in which electronic systems can help avoid the many pitfalls of paper-based systems, the key informants also noted a variety of “value-adds” in electronic systems. For example, respondents noted that information in electronic formats is easier to make available when and where it is needed, allowing for more effective data-driven decision-making:

Data that is reliable, usable, and can allow for decision-making to improve the quality of care and analyze disparities to see who is benefiting from care, who is missing appropriate care, and how the institution can better provide better coverage and quality of care.

[Provider 4]

### Theme 2: Ecosystemic Constraints

#### Overview

Ecosystemic constraints encompass social, political, and economic challenges at a national level that impact the implementation of HISs. These constraints largely exist outside the direct control of a single institution to address.

#### Unreliable Electricity

Every key informant identified insufficient electrical capacity and/or power outages as significant obstacles to the effective functioning of EMRs. As one participant noted:

Well, the national context doesn’t allow for a solely electronic system due to the frequent power outages and internet issues at various independent sites. [...]

[IT or data specialist 8].

In the context of unreliable electricity, participants highlighted another important challenge: a paradox wherein they felt desperate to escape the “trap” of paper-based systems described above but also believed it was impossible to avoid paper at least as a backup because they anticipated frequent electronic system outages due to unreliable power and internet. For example, one key informant noted:

I think we will still use paper because we do not always have electricity, the internet often gives problems, so we will always be able to use paper files. This creates a conundrum for system users who then must spend time on double-data entry in both electronic and paper systems.

[Provider 10]

#### Unstable Internet Access

Along with comments about unreliable electricity, participants also commonly highlighted challenges related to internet access and the inadequate reliability of internet service providers as significant obstacles to implementing reliable electronic systems. They noted that the inability to reliably connect to the internet has direct implications for EMR systems and the degree to which they can rely on state-of-the-art cloud information storage options. As one key respondent noted:

I’m always advocating for more cloud EMRs and the local teams are generally coming back and saying, “that’s not an option for us, our internet is not reliable enough, not fast enough”.

[IT or data specialist 7]

Several health care provider respondents noted that they prefer using the EMR rather than paper, but that internet connectivity (both functioning internet service providers and the stable power necessary to keep internet and EMR platform running) can impede EMR use:

We do have access to the internet, but if the connection is lost, it poses a problem, and we have to write everything down on paper. However, for me personally, the EMR system is easier.

[Provider 1]

#### Unpredictability of the Sociopolitical Landscape

Participants frequently cited social and political instability in the country as a significant barrier to deploying, implementing, and sustaining information systems. For example, one respondent reported:

With political instability and natural disasters, and kind of ongoing safety and security concerns all of those things pose a threat to … a unified EMR and all of these things that we’re trying to work towards from a health information systems perspective.

[IT or data specialist 8]

Although this instability affects the ability to maintain functioning systems, respondents noted how these same social and political contexts also contribute to an increase in the number of patients seeking care at University Hospital of Mirebalais (due to deteriorating conditions at other health facilities), putting even more pressure on the systems:

Initially, we had a hospital that was built to provide patients with specialized care, such as orthopedic, surgical, and trauma care. Now, things have changed with the political problems and the healthcare system, where all patients come to the HUM hospital. Currently, we have almost 700,000 to 800,000 files*.*

[IT or data specialist 5]

### Theme 3: Internal Complexities

#### Overview

This theme highlights challenges that are specific to the organization implementing an EMR. Key informants described how these internal complexities—both technical and operational—could endanger the ability of the electronic system to provide value to clinicians and their patients. Among the IT or data specialists interviewed, there was a strong sense of responsibility to address the complexities and find a way to make the electronic systems more fully meet the end users’ needs. As one respondent described it:

When you implement a system and tell the “provider” that I am simplifying your life, you cannot invent a solution that complicates life. If you invent a solution or propose a […] hybrid system, then you are doing “duplication of tasks” and it will result in more work […]. The quality of data will be altered in both systems, and in the end, you won’t help.

[IT or data specialist 8]

#### Technical Complexities

Several of the challenges mentioned by key informants focused on software development and can be considered “technical” complexities. The first of these is related to integration across multiple existing (and often outdated) systems. Participants reported that current HISs have been deployed for a significant period and are deeply entrenched in the health care system. For example:

It’s like you have a “Stand-alone Application” managing HIV data and you have another “Stand-alone application” managing NCD [non-communicable diseases] data. You have several applications, even in their initial conception, they did not anticipate that they would integrate or connect with other systems. So, when you have these situations, it becomes more difficult.

[IT or data specialist 3]

Due to strong adoption by users and support from stakeholders (including funders, public partners, and users), these systems can be challenging to modify, even when they are not perfectly meeting stakeholders’ needs or when multiple siloed systems would benefit from connecting with each other.

Another technical complexity participants frequently cited is the challenge of developing a master patient index (MPI) across programs and sites within the entire network of care. Respondents flagged that having an MPI is necessary to allow for interoperability and/or integration between systems so that unique patients can be accurately identified across different health care facilities and clinical programs. Developing an optimal MPI would require investments in technology as well as collaboration with the government and other health care delivery organizations nationally to ensure that patient records can follow a patient regardless of where they seek health care. Several respondents noted that the technical challenges in creating an MPI are magnified by the lack of a functional and comprehensive national ID program. Because of the lack of a standard national system to identify unique individuals, investments must be made in other methods to verify identity (such as biometrics):

One of the challenges at the national level, that’s a country-wide challenge, is that there aren’t many legal documents to allow you to identify patients or access their services. […] you know that there’s a part of the population that doesn’t even have a birth certificate, let alone other official identity pieces like a National ID Card or passport.

[IT or data specialist 6]

A final type of technical complexity mentioned by key informants relates to the science and art of “requirements gathering” or the tailoring of a software solution to meet all the needs of its varied users. Participants commonly expressed concerns regarding information systems that fail to consider the needs of users and the workflow of health care operations. Due to gaps in the design process, these systems often do not comprehensively incorporate user input, leading to poor user experiences and a lack of solutions tailored to meet real-world needs. Respondents described a growing awareness that more effort must be spent on understanding all nuances of the clinical workflow so that the HIS matches those processes. As one respondent noted:

[If] it is not “user-friendly,” it is also because the logic of the provider’s trade does not reflect it. [...]. So, when you develop a system, you must have a process that allows you to approach your solution as closely as possible with its work and the usual task flow.

[IT or data specialist 8]

#### Logistical and Operational Complexities

Even after an electronic system has been fully built, there are logistical and operational complexities that can impede optimal use. One of the most common operational challenges noted by key informants was related to user support. Participants noted that insufficient staffing to provide on-site support during the deployment of information systems is a common obstacle that undermines successful system implementation. When doctors and nurses encounter technical problems while using the system, they may struggle to find immediate assistance, which can lead to delays in care provision and lower uptake of the system:

When you have a line of patients waiting for you to provide service, the system must be able to perform efficiently, so that when you click on a button, it doesn’t take 15 to 20 seconds. It must be performant enough to free up your line as much as possible.

[Provider 3]

Key informants noted that this challenge of user support is affected both by financial resource constraints and by limitations in the ability to find and retain technical staff willing to work at the point of care in the many rural facilities supported by ZL or Partners In Health:

You need the money but it’s a little bit easier to hire software developers who can work remotely. You know whether it’s in Haiti, remotely, or in Boston to develop software. But it’s hard, it’s a little bit harder to hire people who are going to every site […]. Then, to train all the users continually, […] be there to troubleshoot the system with the users, […] monitor the use of the system to make sure it’s being used properly and then to monitor that the data is being extracted properly, and […] analyzed properly. That’s, I think, where we struggle the most.

[IT or data specialist 8]

Even beyond the specific issue of finding and retaining human resources for point-of-care user support, participants also flagged the overarching issue of insufficient allocation of financial resources to information management overall. They reported that the lack of dedicated funding translates into an inability to upgrade IT infrastructure equipment, acquire or retain skilled human resources, and other necessary investments that are critical for effective information management. One respondent noted that this underresourcing may stem from the iterative process by which electronic information systems often evolve:

...But now we need to transition to an electronic system. So, it’s already an interactive activity and is being implemented gradually, not completely or integrally. Therefore, there is no specific fund dedicated to it, so that you can say in one or two years, you have 2 or 3 million dollars for it. Because the information system is not like that. It is progressive and with the available resources, you will advance with the strategic direction you want.

[IT or data specialist 9]

The final major operational complexity mentioned by key informants was competing priorities from varied stakeholders, including funders, care providers, and public partners. The key informants identified these competing priorities as significant factors contributing to siloed EMRs, system duplication, and the evolution of an increasingly incoherent and complex information system landscape over time. The competing demands often result in the addition of new parameters to existing systems or the development of parallel systems to meet specific requirements, which can create complexity and hinder the interoperability of information systems. As one respondent noted:

Sometimes a partner may ask for a certain parameter, while another partner may ask for a different parameter, and it can be difficult to satisfy both partners.

[IT or data specialist 8]

Respondents also reflected that the growing complexity in the information system landscape is a natural consequence of the incremental nature of system improvement and the need to continually evolve to meet changing needs:

We have some challenges that we face every day, we have an information system that does not necessarily respond to all our needs, so we gradually have to patch it to conform to new demands.

[IT or data specialist 6]

### Theme 4: Building Resilient Information Systems

The final theme is focused on participants’ insights and aspirations for improvements that could build more robust information systems in the future, overcome current challenges, and deliver greater value to their users.

#### Prioritizing Continuous System Access

Participants expressed a desire for the development of electronic systems that can be continuously accessed, even when an internet connection is unavailable. Specifically, they hope for systems that can function offline and be connected to a centralized master patient index. This would enable continued use of a system even when there is no internet connection, with local data subsequently synchronized to a broader, cloud-based system once an internet connection becomes available again. The participants emphasized that mitigating connectivity challenges while also enabling integration is key to greater system usability. As one provider noted:

One of the requirements I would make is to ask how I can have an offline version of this application. This means that people would be working on the cloud, but there would be synchronization that could be done offline, which would allow for problems with the internet ... or people could work offline but when there is power or internet, it would replicate online.

[Provider 4]

#### Right-Sizing the Paper Footprint

Key informants hope for the development of an information system that is less dependent on paper. However, their ambitions are diverse in scope. Some contend that a paperless system can be realized if adequate resources are invested, while others suggest that physical files should still function as backup in the event of electronic system failure. The following quotations illustrate these differing perspectives:

I think we should keep in mind that we have very few barriers to paperless. [...] I would love to see us come up with a really great interface on tablets, so that we could replace paper. And then, there’s no risk of delays and data entry errors and all that. I mean, of course, you can still have data entry errors on tablets. But yeah, I think we could do that*.*

[IT or data specialist 7]

[...] There are always other problems that come up, there are always outages, so we can’t guarantee it 100%. The risk that exists is if we can’t access the electronic file, then what? Will we never see that file again, or will we just take an old file and put it on paper? That’s the dilemma.

[IT or data specialist 8]

#### Integrated Electronic Medical Records

The respondents commonly expressed the need for a unified patient record system that facilitates the access and sharing of extensive patient information by health care providers in real-time. This objective is believed to enhance patient care coordination and ultimately optimize patient outcomes. Both clinical providers and IT or data specialists shared these aspirations for how better systems might help achieve better service provision to patients. For example:

[…] it’s important that we have a single electronic system for any patient, because remember the patient’s medical history is their story, so if I’m giving the patient care, I have to have access to their history, even with their child’s medical history, they were diagnosed with such a problem at such a year, but what was done for them. If they had an allergy to such a medication, I’ll know the medication I’m not allowed to give them, they had an allergy at such a time ...

[Provider 4]

#### Prioritizing Enterprise Resource Planning

Even beyond the common aspirations expressed by most key informants, one participant articulated a more comprehensive future vision, focusing on the necessity of extending system integration beyond patient records. They emphasized the importance of prioritizing the integration of other administrative functions, such as human resources, procurement, finance, and operations. The aim of such integration is to enhance the efficiency of the services that support care delivery within hospitals. This vision and perspective are clearly articulated in the following quotation:

Today, if you go to the Purchasing or HR department, there is no application for managing their business processes. There is no application that manages inventory resources, no application that manages purchasing, logistics, and many others. The majority of non-clinical professions do not have applications that ensure the management of their processes. [...] One of the well-understood projects is to come up with an ERP [Enterprise Resource Planning system], a type of software that is fully integrated. [...] And this ERP would have an API [Application Programming Interface] that could allow us to integrate it with other existing applications at the system level.

[IT or data specialist, Haiti 9].

## Discussion

### Overarching Findings

The research findings suggest that the long-term sustainability of digital information systems, such as EMRs, in fragile, resource-constrained settings depends not only on modern innovative solutions but on the broader ecosystemic constraints [13,14] and internal complexities in which these systems are embedded. This finding is congruent with a system-theoretical perspective, as it points to the necessity of a holistic approach to analyzing interconnected systems [15-17], in particular the sociotechnical analytical framework, which theorizes the complex interplay of technology, users, workflows, and institutional environments [15] to understand technological change. It also reveals that, despite many existing challenges, stakeholders perceive strong promise in digital systems to help overcome the serious limitations inherent in paper-based systems and to improve patient care. More importantly, the results of this study suggest several protective strategies that, if implemented well, can increase long-term EMR resilience and enhance their maintainability in such fragile contexts.

### Mitigating Multi-Cascading Failures Across Interdependent Systems

The concept of resilience in HISs presupposes, by definition, the ability of the information system to withstand disruptions and recover core functionality after failure [15,18,19]. Although digital system integration offers important potential benefits for the continuity of care, this continuity may be undermined by multi-cascading failures from interdependent systems [20-22]. In resource-constrained settings, inadequate national infrastructure is a recurrent challenge [3,8,13,23-28]. As observed in this study, the literature on electronic health system implementation in comparable contexts consistently highlights the constraints imposed by unreliable interdependent infrastructure [14,23]. In such settings, the occurrence of disturbances affecting interdependent systems, particularly electricity and internet connectivity, is often inevitable. Concurrently, the joint optimization of technical and social parameters is rarely attainable, given the significant resource investments required and the overarching scope of such interventions. From a theoretical perspective, the frequent occurrence of disruptions destabilizes the established resilience theoretical framework distinction of pre- and postdisaster phases and suggests the need for a new perspective that accounts for the state of permanent crisis in such contexts. Thus, there is a need for devising mitigation strategies to strengthen HIS resiliency.

The content analysis of the interview transcripts suggests an implicit consensus among participants on the need to prioritize enhancing the robustness and redundancy attributes of HIS resilience. Participants indicate that resiliency can be strengthened by building redundancy into power sources on premises to mitigate reliance on unstable public uses and diversifying connectivity sources including the adoption of emerging technologies, such as Starlink low-earth orbit satellite internet, which may constitute a viable solution in infrastructure-constrained settings. Respondents also described the need to strengthen the robustness attribute through proper routine maintenance of on-premises IT and power infrastructure, as well as through a hybrid information system topology with right-sized paper footprint serving as a buffer component to EMR failure. The literature on interdependent infrastructure resilience consistently identifies redundancy and robustness as foundational attributes for improving system resilience [29-31].

Although redundancy in power sources remains essential, viable alternatives to many on-premises IT infrastructure do exist, particularly through the adoption of cloud-based solutions. However, this transition comes with an important trade-off: the reduction in costs associated with IT infrastructure maintenance and specialized IT staff salaries comes with an increased dependency on reliable electricity and internet connectivity.

### Perceived Promise of Electronic Systems

A key component of the perceived promise of electronic systems stems from the obvious untenability of paper-based information systems [32,33]. The limitations of physical storage, as paper files necessitate space that cannot be perpetually expanded [34,35], contribute to suboptimal management of patient records, with potentially consequential implications for the continuity and quality of care delivered to patients. Participants suggested that the gradual dematerialization of paper records through systematic scanning may offer a pragmatic pathway toward reducing the paper footprint of the health information system. Extending this perspective, contemporary AI-enabled cloud solutions, such as Azure AI Document Intelligence and native AI document parser in the Databricks platform, may provide relatively affordable and scalable means for accelerating the digitization, extraction, and processing of information contained in scanned records [36,37]. However, the practicality of such solutions in resource-constrained settings hinges on the ability to fully transition to modern, cloud-based enterprise resource planning infrastructure, a shift that may increase dependence on unstable interdependent systems: electricity and connectivity. A paradox emerges, wherein paper-based systems are perceived simultaneously as a problem to be addressed through the transition to EMR and as a necessary stop-gap solution during gaps in power or connectivity.

Moreover, another perceived promise of modern EMRs lies in their ability to support continuity of care along the patients’ care pathway across time and space. Central to this process is EMR “interoperability,” defined as the capacity of diverse EMR subsystems within the broader HIS macro-system to exchange, interpret, and use information coherently and seamlessly. Interoperability therefore constitutes a foundational mechanism of integration, through which otherwise distinct digital components are functionally connected to support coordinated system performance and broader health system objectives. In the context of the 2 public health facilities included in this study, the semantic layer of EMRs aligns, to some extent, with known standards such as SNOMED CT (Systematized Nomenclature of Medicine–Clinical Terms) for clinical terminology and LOINC (Logical Observation Identifiers Names and Codes) for laboratory testing, but the inability to uniquely identify patients remains a significant barrier to interoperability. Therefore, achieving effective interoperability depends on an underlying MPI that can reliably establish and maintain patients’ identities across time and place, thereby supporting EMR integration across vertical programs and among health care institutions over time [13,32,38-40].

Existing literature originating from studies in low-resource settings emphasizes both the significance of an MPI in achieving interoperability and the challenges in making MPI a reality. For instance, a study conducted in Nigeria highlights the challenges associated with the development of a reliable MPI, as no current strategy satisfactorily fulfills the requisite attributes (uniqueness, unchanging, uncontroversial, inexpensive, ubiquitous, and uncomplicated) for dependability [13,32,38-40]. These concerns are particularly pertinent in the context of Haiti, where the national identification system is unreliable and incomplete, especially among the most impoverished citizens—a common challenge in resource-constrained contexts [26,38,41].

While it is easy to imagine “digital transformation” as a linear progression from paper-based systems to basic electronic systems and ultimately to electronic systems with increasingly sophisticated functionality, this research found that “legacy” systems create substantial internal complexities that can in fact be an obstacle to improving HISs. These legacy systems or vertical, disease-specific EMRs tend to dominate HISs in LMICs [42]. The complexities engendered by these legacy systems stem from concerns related to their initial design and underlying technologies, which cannot be adequately remedied through patchwork modifications. Simultaneously, a comprehensive revamping of these legacy systems necessitates substantial financial and human resources [6,43] that may not be immediately available and could be perceived as a risky place to invest.

### Protective Strategies

The results of this study suggest several protective strategies that could help implementers increase EMR resilience. First, a strong focus on the integration of systems is a strategy that can address the internal complexities related to legacy systems [38,44]. While full-scale replacement of siloed systems—which are often managed by disparate partner organizations—may not be feasible, investment in initiatives to connect systems through application programming interfaces, streamline terminologies, or otherwise facilitate data exchange and interpretability between systems may help users access the information they need and reduce the need for error-prone duplicate data collection across multiple platforms [8].

A second protective factor involves a stronger focus on requirements for EMRs to ensure better alignment with existing clinical workflows, support clinicians [45,46], and limit the duplicated data collection burden that poorly align with patient care processes [47,48]. This necessitates prioritizing viable solutions that address existing challenges without introducing further complications. As aptly articulated by one participant: “when you implement a system and tell the ‘provider’ that I am simplifying your life, you cannot invent a solution that complicates life [...].”

Finally, the findings underscore the importance of adopting emerging technological innovations that may alter the conditions for EMR implementation in sudden and positive ways. Innovations such as low-Earth-orbit satellite internet service may help mitigate persistent infrastructure constraints characteristic of LMICs.

This study bridges 2 strands of sociotechnical literature: one concerned with the historical, contingent, and path-dependent dynamics of technological transitions and another focused on the configurations of conditions that enable such transitions toward more sociotechnical equilibria [49]. Drawing on empirical evidence of EMR implementation in Haiti, the findings show that digital HIS transformation in fragile and resource-constrained settings cannot be understood as a discrete act of technology adoption. Rather, it is a sociotechnical transition shaped by ecosystemic constraints, internal complexities, and resilience strategies that determine whether EMRs can become durable components of care delivery.

### Limitations

This study focused on EMR implementation at 2 public health care facilities supported by ZL in Haiti. Although the findings provide important insights into EMR sustainability in resource-constrained settings, they should be applied with caution when considering applicability and generalizability to other health care institutions in Haiti or to other LMIC contexts. ZL-supported facilities may benefit from additional technical, financial, infrastructural, and organizational resources that are not consistently available in public facilities funded primarily through local or national governmental mechanisms.

Furthermore, the research did not examine the perspectives of critical stakeholders from the Haitian Ministry of Health, who could provide critical insights into how donor-state dynamics shape broader health information policies, governance structures, and priorities related to HIS. This is a critical gap that affects the generalizability of the findings.

Finally, community outreach is increasingly becoming a cornerstone of care delivery in LMICs where access to facility-based care remains limited [50-53]. This study did not examine existing information systems used by community health programs, nor did it assess their potential role in improving the continuity of care through the linking of facility-based care with community-level service delivery.

### Conclusions

This study adds to a growing base of evidence shedding light on key factors that support or endanger the success of digital transformation for HISs in resource-constrained settings. The study findings underscore that the successful implementation of EMR for improved clinical care necessitates a comprehensive strategy, considering both the ecosystemic constraints at the national level and the internal complexities within the existing system implementation contexts. It also highlights a set of protective strategies that sustain and improve EMR resilience in fragile and resource-constrained settings. These strategies include prioritizing integration and interoperability between systems, building redundancy to mitigate cascading failures derived from other interdependent systems, strengthening staffing to support system use, right-sizing the paper footprint (eg, improving handwriting-to-text scanning and digitization solutions), and embracing technological innovations.

## Abbreviations

EMR: electronic medical record
HIS: health information system
HUM: Hôpital Universitaire de Mirebalais
IRB: institutional review board
LMIC: low- and middle-income country
LOINC: Logical Observation Identifiers Names and Codes
MPI: master patient index
MSPP: Haitian Ministry of Health
PI: principal investigator
SNOMED CT: Systematized Nomenclature of Medicine–Clinical Terms
ZL: Zanmi Lasante
